# When better data meets better design: How EHR data usability and system usability shape physicians’ cognitive load

**DOI:** 10.1038/s41746-025-02243-4

**Published:** 2026-01-14

**Authors:** Curtis A. Merriweather, Jr., Kalle Lyytinen, David Aron, Michael R. Cauley

**Affiliations:** 1 Fuqua School of Business, Duke University, Durham, NC USA; 2 Weatherhead School of Management, Case Western Reserve University, Cleveland, OH USA; 3 Department of Biomedical Informatics, Vanderbilt University Medical Center, Nashville, TN USA

**Keywords:** Software, Technology, Computational platforms and environments

## Abstract

Electronic health record (EHR) systems were designed to enhance clinical decision-making, yet the way data is organized and displayed can create significant cognitive demands for physicians. This study examines how EHR *data usability* (data quality, data completeness, and data-driven use) and *system usability* jointly shape physicians’ cognitive load. Using survey responses from 564 physicians across 32 specialties, we tested a mediated model with covariance-based structural equation modeling. Reliability and validity were assessed through standard psychometric criteria. Findings show that stronger data usability increases germane cognitive load, promoting deeper engagement with clinically meaningful information. In contrast, higher system usability reduces extraneous cognitive load by aligning interface design with clinical workflow and minimizing navigation-related effort. Information overload partially mediated these effects, suggesting that better data usability helps physicians better filter irrelevant data and stay focused on diagnostically relevant cues. Overall, the results highlight two levers for improving cognitive performance: enhancing system usability lowers unnecessary cognitive effort and documentation-related errors, while improving data usability supports reasoning-intensive diagnostic work. Optimizing both fosters balanced cognitive load and more sustainable, error-resilient clinical decision-making.

## Introduction

Electronic health record (EHR) systems are computer-based information systems that capture and maintain patients’ health data, including events, treatments, and care activities^1^. Beyond serving as repositories of clinical information, EHRs underpin essential organizational functions for billing, workflow coordination, regulatory compliance, and clinical research^1,2^. As patient data volume and complexity continue to grow, physicians increasingly depend on EHR systems to document, retrieve, and share information across care teams. This dependency has shifted core aspects of clinical reasoning and decision-making into EHR-mediated workflows. Emerging evidence shows that these activities introduce substantial cognitive load, particularly after nurse intake and the initial patient-physician encounter for established patients^3,4^. Consequently, as EHR use becomes more deeply embedded in physicians’ work, EHR-related factors exert growing influence on the quality, efficiency, and outcomes of patient care decisions^5^. EHR systems are widely expected to improve healthcare quality and performance by increasing the availability and accessibility of patient data^6^. However, empirical studies consistently show that greater EHR use does not automatically translate into improved care quality^6^. The relationship between EHR data quality and patient care outcomes is more nuanced, requiring closer attention to how physicians use EHR data and to the extent to which their EHR use aligns with clinical workflow demands and contributes to cognitive load^7^. Cognitive load, in this context, reflects the mental effort required for physicians to identify, maintain, and use EHR-based patient information with other relevant cues during patient care.

One explanation for the modest impact of EHRs on care quality is the difficulty of using these systems during routine clinical work. Poor data organization, suboptimal information presentation, and complex interfaces often make it challenging for physicians to identify clinically salient information, while extensive documentation requirements demand continuous attention^8–10^. These sociotechnical challenges persist even with the introduction of digital scribe technologies, underscoring the need to differentiate between the EHR as a data infrastructure and the user interface (UI) through which physicians interact with that infrastructure^11^. Cognitive overload does not stem from the underlying database architecture, but rather from interface complexity, navigation burden, and misalignment between the system workflows and clinical reasoning processes. A national study of 8274 U.S. physicians reported a mean EHR usability score of 45.9 on the System Usability Scale, equivalent to a failing grade (“F”), which reflects widespread dissatisfaction with EHR design and workflow integration^12–15^. The difficulty of using EHR systems during clinical work has even been likened to texting while driving^16^. This raises concern about the potential risks posed by high cognitive demands. Several studies report substantial adverse effects. For example, a recent study suggests that EHR systems provide limited support for core cognitive tasks, impose significant cognitive load^3,17^, and can hinder physicians’ ability to process patient information and engage in higher-level reasoning^18^. As EHRs accumulate increasingly large volumes of structured and unstructured data, physicians face additional challenges in finding relevant information, resulting in information overload and further elevating cognitive load^19–21^. Recent work in npj Digital Medicine further emphasizes that physicians face persistent information overload, requiring advanced filtering or design interventions^22^. Taken together, these findings suggest that even as EHR data quality and completeness improve, the processes required to access and use that data may increase cognitive load and exacerbate information overload^23^. In this paper, we ask: to what extent does EHR use in a clinical setting affect cognitive load, and specifically, to what extent related factors of system usability and EHR information overload influence physicians’ cognitive load? To address this research question, we first review EHR systems within a sociotechnical framework, focusing on how information overload and system usability shape clinical cognition^24–28^. Next, we develop a mediated research model that incorporates EHR data usability, EHR system usability, and information overload, and propose four hypotheses describing their combined effects on physicians’ cognitive load. We evaluate this model using survey data from 564 practicing physicians who use EHR systems. Finally, we present the key findings, outline their implications for theory and practice, and identify directions for future research.

This study explores how EHR system usability and data usability together affect physicians’ cognitive load during clinical decision-making. Using cognitive load theory as the framework, we develop and empirically test a model to explain how interface-driven system usability and the usability of clinical data contribute to distinct forms of cognitive effort, including extraneous and germane load. This work extends our preliminary model by providing a more granular account of the cognitive mechanisms activated during EHR-mediated clinical reasoning^29,30^.

## Results

### Participant characteristics

A total of *N* = 564 physicians representing 32 specialties, from Family Medicine to Thoracic Surgery, completed the survey (see Fig. 2).

**Fig. 1:**
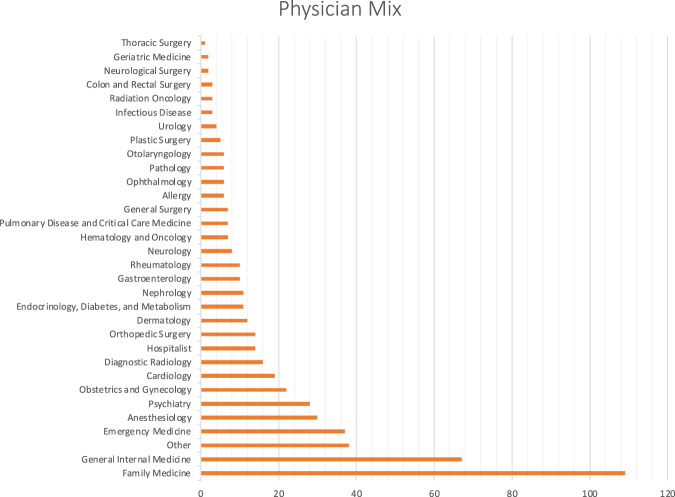
Surveyed physician specialty distribution. This figure shows the distribution of surveyed physicians across medical specialties. Bars represent the number of respondents in each specialty category, illustrating the breadth of clinical representation in the sample, from primary care to surgical subspecialties.

Overall, the sample included physicians from 35 medical specialties, reference Fig. 1.

The majority reported using one of the major commercial EHR systems, Epic (51.6%), Cerner (12.2%), Allscripts (6.6%), or Meditech (5.7%), representing the predominant platforms used in U.S. healthcare delivery, reference Table 1. All participants reported experience with at least one major commercial EHR platform. Most identified use of leading systems such as Epic, Cerner, and Allscripts, which share standard features including computerized provider order entry (CPOE), clinical documentation modules, and integrated clinical decision support.

**Table 1:** EHR system use

	EHR system	Count	User percentage
1	Allscripts	37	6.6%
2	Cerner	69	12.2%
3	Epic	291	51.6%
4	Meditech	32	5.7%
5	Other	112	19.9%
6	None	23	4.1%
	Total	564	100%

Demographic variables included age, gender, specialty, and years of EHR use. Table 2 presents descriptive statistics for participant characteristics. General Practice and Internal Medicine together accounted for 33.6% of respondents. Participants’ clinical experience clustered primarily within the mid-to-late career range, with the largest portion (18.1%) reporting 21–25 years of practice. This distribution supports the reported mean experience score of 4.84 on the categorical scale used. EHR system use patterns aligned with national trends: 51.6% of respondents’ active use of Epic, consistent with descriptive item responses. Most participants practiced in organizations where Epic was the primary system. This distribution corresponds to the observed mean of 3.21 for EHR platform use. Ethnicity and gender were moderately skewed, reflecting the demographic composition of the sample. White physicians represented 70.6% (skew = 1.452). Male physicians comprised 66.3% of respondents (skew = 2.046), with the remainder identifying as women and/or choosing not to disclose. Office-based practice was the most common setting (51.27%), consistent with its mean value of 3.42 on the categorical scale. Collectively, the descriptive statistics indicate that the sample was characterized predominantly by mid-career male physicians practicing in office-based settings and using the Epic EHR system (see Table 2).

**Table 2:** Statistical analysis of participant demographic data

	Range	Minimum	Maximum	Sum	Mean		Std. deviation	Variance	Skewness		Kurtosis	
	Statistic	Statistic	Statistic	Statistic	Statistic	Std. error	Statistic	Statistic	Statistic	Std. error	Statistic	Std. error
Education	2	1	3	981	1.91	0.016	0.366	0.134	−1.108	0.108	3.510	0.215
EHR system	4	1	5	1650	3.21	0.049	1.122	1.258	0.170	0.108	−0.389	0.215
Ethnicity	6	1	7	1096	2.13	0.085	1.929	3.721	1.452	0.108	0.734	0.215
Gender	3	1	4	713	1.39	0.028	0.634	0.401	2.046	0.108	5.332	0.215
Medical specialization	34	1	35	6226	12.11	0.449	10.181	103.660	0.972	0.108	−0.405	0.215
Place of practice	7	1	8	1759	3.42	0.082	1.857	3.449	1.032	0.108	0.411	0.215
Years of experience	9	1	10	2488	4.84	0.087	1.982	3.928	0.161	0.108	−0.744	0.215

#### Exploratory factor analysis (EFA)

The final solution yielded 6 theorized factors comprising 19 indicators and demonstrated strong psychometric adequacy (KMO = 0.895; Bartlett’s test *p* = 0.00 and commonalities > 0.34). All retained factors had eigenvalues greater than 1 and together explained 73.27% of the total variance. Internal consistency was acceptable for all factors (Cronbach’s *α* > 0.70). Convergent validity was supported by item loadings above 0.422 (Hair et al.^31^), and discriminant validity was demonstrated by the absence of cross-loadings above 0.30 and factor correlations remaining below 0.70.

#### Confirmatory factor analysis (CFA)

The CFA supported a four-factor structure with 19 items, with only one minor exception related to three social desirability items. Overall model fit was acceptable according to recommended criteria (Hu and Bentler^32^): *χ*^2^ = 389.955, df = 196, CFI = 0.967, TLI = 0.958, RMSEA = 0.044, PCLOSE = 0.942, and SRMR = 0.041. Reliability and validity metrics were acceptable (reference Table 3), with all constructs demonstrating average variance extracted (AVE) > 0.50 and composite reliability (CR) > 0.70. Discriminant validity criteria were met across all factors, reference Table 3.

**Table 3:** Convergent and discriminant validity table

		CR	AVE	MSV	ASV	Cognitive load	Data completeness	Data driven	Data quality	EHR usability	Information overload
	Cognitive load	0.760	0.514	0.176	0.026	**0.717**					
EHR data usability (second-order formative factor components)	Data completeness	0.861	0.676	0.506	0.235	−0.162*	**0.822**				
	Data driven	0.715	0.558	0.138	0.083	0.074	0.372*	**0.747**			
	Data quality	0.871	0.536	0.517	0.216	−0.212*	0.705*	0.326*	**0.732**		
	EHR usability	0.894	0.737	0.517	0.249	−0.329*	0.711*	0.367*	0.719*	**0.859**	
	Information overload	0.817	0.690	0.221	0.126	0.42*	−0.444*	−0.056	−0.417*	−0.47*	**0.831**

*p < 0.05.

#### Common latent factor (CLF) and multicollinearity

A common latent factor (CLF) adjusted model demonstrated poor model fit: *χ*^2^ = 771.384, df = 132, CFI = 0.872, TLI = 0.834, RMSEA = 0.097, PCLOSE = 0.000, and SRMR = 0.452 (Hu and Bentler^32^). Model fit indices indicated poorer fit under the CLF-adjusted model. Variance inflation factors (VIFs) were below 5.0 for all constructs, indicating no issues with multicollinearity.

#### Structural model

The final structural model demonstrated excellent fit: *χ*^2^ = 15.993, df = 10, CFI = 0.995, TLI = 0.979, RMSEA = 0.048, PCLOSE = 0.477, and SRMR = 0.036^32^. Multiple *R*^2^ values indicated that EHR data usability accounted for substantial variance in cognitive load (34.1%), EHR usability (80.4%), and information overload (29.2), reference Table 4. Figure 3 presents the final hypothesized structural model in this study, showing the hypothesized relationships among EHR data usability, system usability, information overload, and cognitive load.

**Table 4:** Direct effect regression model results

Standardized (*β*) coefficients			
Variables	Estimate	S.E.	*p* value
Cognitive load ← EHR data usability	0.597	0.125	0.000
Information overload ← EHR usability	−0.262	0.112	0.019
*R*-square			
Cognitive load	0.341	0.052	0.000
EHR usability	0.804	0.021	0.000
Information overload	0.292	0.054	0.000

### Hypothesis testing

**Fig. 2:**
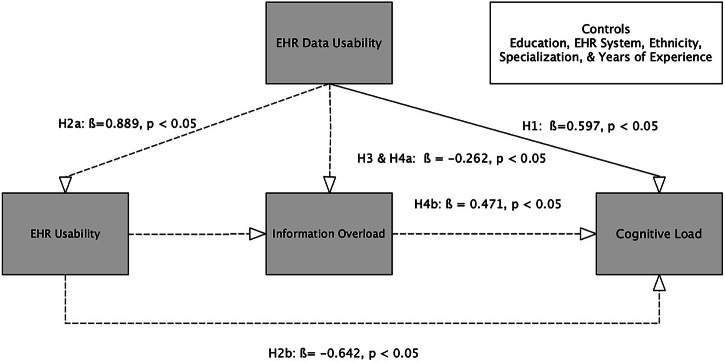
Final structural model with standardized path estimates. This figure presents the final tested structural equation model, showing standardized path coefficients (*β*) for all significant relationships among EHR data usability, system usability, information overload, and cognitive load. Solid arrows indicate statistically significant paths (*p* < 0.05), and the direction of effects reflects hypothesized positive or negative associations. The model illustrates both direct and indirect (mediated) effects contributing to physicians’ cognitive load during EHR use.

Concerning EHR system use, more than half of respondents reported using Epic (51.60%), followed by Cerner (12.23%), Allscripts (6.56%), and Meditech (5.67%), reference Table 1. Table 4 summarizes the results of the direct effect regression model, and Table 5 summarizes the mediation results for the final structural model. A strong direct effect was observed between EHR data usability and cognitive load (*β* = 0.597, *p* = 0.000***), supporting Hypothesis 1 (Tables 4 and 6). For Hypothesis 2, EHR system usability partially and negatively mediated the relationship between EHR data usability and cognitive load. The indirect path from EHR data usability → EHR system usability → Cognitive load was significant (*β* = −0.571, *p* = 0.019*), providing support for H2 (Tables 5 and 6). The direct effect of EHR system usability on information overload was also significant (*β* = −0.262, *p* = 0.000***), supporting Hypothesis 3 (Tables 4 and 6). For Hypothesis 4, the mediated pathway from EHR data usability → information overload → Cognitive load was significant (*β* = −0.137, *p* = 0.014*), providing additional support for the hypothesized model (Tables 5 and 6). A visual summary of all hypothesized paths and their significance levels is presented in Fig. 2.

**Table 5:** Mediation results

Direct, indirect, and total effects				
	Mediation	EHR data usability		
		Direct	Indirect	Total
EHR usability			−0.571*** [−0.783 −0.363]	−0.816*** [−1.031 −0.605]
Information overload			−0.124* [−0.261 −0.023]	
EHR usability and information overload			−0.122* [−0.229 −0.033]	
Cognitive load		0.597*** [0.359 0.841]		0.597*** [0.359 0.841]
	**Mediation**	**EHR usability**		
		**Direct**	**Indirect**	**Total**
Information Overload			−0.137* [−0.257 −0.037]	−0.137* [−0.257 −0.037]
Cognitive Load		−0.642*** [−0.876 −0.405]		−0.642*** [−0.876 −0.405]

Brackets show 95% confidence intervals.

*p < 0.05, **p < 0.01, ***p < 0.001 two-tailed, *N* = 513.

**Table 6:** Hypothesis table

Hypothesized relationships		Supported (Y/N)	Evidence
Hypothesis 1	EHR data usability has a direct and positive effect on Cognitive Load	Fully Supported	*β* = 0.597, *p* < 0.05
Hypothesis 2	EHR usability partially and negatively mediates the positive relationship between EHR data usability and cognitive load.	Fully Supported	*β* = −0.571, *p* < 0.05
Hypothesis 3	EHR data usability directly and negatively influences information overload.	Fully Supported	*β* = −0.262, *p* < 0.05
Hypothesis 4	The positive effect of EHR data usability on cognitive load is negatively and partially mediated by information overload.	Fully Supported	*β* = −0.124, *p* < 0.05

All controls were nonsignificant with one exception: years of experience were negatively associated with cognitive load (*β* = −0.145, *ρ* = 0.005**), indicating that more experienced physicians reported lower cognitive load.

## Discussion

The findings of this study offer new insight into how EHR system usability and EHR data usability jointly influence physicians’ cognitive load during clinical decision-making. Consistent with cognitive load theory, the results indicate that EHR system usability is negatively associated with extraneous cognitive load through its effects on navigation, interface clarity, and workflow alignment. In contrast, EHR data usability enhances germane cognitive load by increasing the quality, relevance, and completeness of information available for clinical reasoning. This study advances prior literature by integrating data quality and interface usability within a cognitive load theory framework. Whereas earlier work typically examined UI design or information presentation in isolation, our model empirically demonstrates how both dimensions interact to shape physicians’ cognitive effort. This dual-construct perspective highlights that effective EHR design must balance the quality of available information (germane load) with the efficiency of system interaction (extraneous load), thereby supporting more cognitively sustainable digital clinical environments.

EHR usability has long been recognized as a barrier to high-quality patient care^33^. Physicians consistently report misaligned workflows, excessive documentation requirements, and unintuitive interfaces as major contributors to cognitive burden. Collectively, these design shortcomings create friction between clinical reasoning and system interaction^34,35^. Although the EHR functions as a repository for patient data, cognitive overload arises primarily from how information is accessed and navigated through interface design, data presentation, and workflow integration, rather than from the underlying data structures themselves. Clarifying this distinction helps identify where design interventions should be directed. A sociotechnical perspective further underscores how data flows, particularly those involving patient-generated data, shape physicians’ cognitive burden^36^. Common EHR usability pain points include:

- Alert fatigue: frequent, low-signal warnings that draw attention away from critical tasks
- Click burden: excessive navigation steps that interrupt clinical flow
- Workflow misalignment: system functions that fail to reflect real-world care processes
- Visual clutter: poorly organized screens that obscure diagnostically relevant patient information^3^

Addressing these issues through human-centered re-design, consistent interface logic, and tighter workflow integration can substantially reduce extraneous cognitive load and improve decision-making efficiency. Emerging collaborative intelligence approaches may further reduce documentation and navigation demands by distributing cognitive work across human-artificial intelligence (AI) teams^37^. As EHR data usability improves, physicians can more readily identify and prioritize clinically relevant patient information while ignoring redundant or low-value content. This aligns with prior evidence suggesting that higher-quality, context-relevant data enables physicians to focus on diagnostically meaningful cues and filter out noise^38^.

From a cognitive load theory perspective, the relationship between EHR data usability and cognitive load should not be viewed as purely reductive. Although improved data usability is associated with lower perceived information overload, this reflects a rebalancing of cognitive load rather than a reduction in its overall magnitude. Higher-quality, contextually relevant data increases germane cognitive load, prompting deeper analytic reasoning and more effective diagnostic interpretation. In parallel, enhancements in interface usability and workflow alignment reduce extraneous cognitive load by decreasing the mental effort required for navigation, documentation, and system interaction. Together, these shifts produce a more balanced and sustainable cognitive load profile, one that enables physicians to focus their cognitive resources on clinical reasoning instead of compensating for technological friction.

Translating these theoretical insights into practice requires identifying the conditions and design levers that enable a balanced cognitive workload for physicians. The findings of this study operate within specific boundary conditions but point to several actionable opportunities for reducing cognitive imbalance. First, extraneous cognitive load can be minimized through targeted UI refinements, such as prioritizing high-signal alerts, consolidating task flows to reduce navigation steps, and collocating key diagnostic information within a single view. These adjustments reduce unnecessary cognitive switching and better align visual hierarchies with clinical reasoning^39^. AI-enabled interfaces that learn from user interaction patterns may further streamline workflows and reduce cognitive burden. Second, data governance mechanisms are essential to ensuring high-quality information without overwhelming users. Strategies such as suppressing duplicate data entries, embedding provenance indicators to signal reliability, and standardizing data completeness rules can strengthen germane cognitive engagement while preventing data redundancy or noise. Together, these levers outline the practical conditions under which “better data” and “better design” converge to support a cognitively sustainable digital clinical environment. Because this study examined U.S.-based physicians using major commercial EHR platforms, the findings should be interpreted within this context^40^. Although many core usability and data quality issues are broadly applicable, differences in EHR design, localization, regulatory requirements, and adoption patterns across international health systems may shape cognitive load in distinct ways.

The effects of information overload and EHR use on cognitive load are nuanced. Our results indicate that both factors independently influence physicians’ cognitive burden. When cognitive load becomes excessive, the quality of clinical decision-making declines, underscoring the need to manage both information overload and EHR interaction demands. Achieving this balance requires thoughtful user-centered design and tight workflow integration; without these, physicians’ cognitive load will continue to exceed sustainable levels. Optimizing EHR usability begins with ensuring that patient data captured during clinical workflows are complete, high-quality, and clinically meaningful. Such data must also be easily findable, actionable, and practical for real-time decision support. Accordingly, evaluating EHR system use should be treated as a diagnostic process to identify where data effectiveness, work efficiency, and interface design succeed or fall short within an organization. These diagnostic insights must then inform targeted interventions that enhance perceived system usability. Although many factors influence EHR system usability, three key opportunities emerge:

1. Applying human factor re-design principles to align interfaces and workflows with physicians’ cognitive processes.
2. Ensuring consistency within and across applications, reducing variability that disrupts workflow and increases cognitive switching.
3. Reexamining data effectiveness and data-use practices^41^, promoting structured, reliable, and contextually meaningful information for clinical work

Multiple paths exist to improve EHR usability, but meaningful progress will require disruptive innovation that prioritizes user-friendly, physician-centered design with an integrated system architecture^23^.

Cognitive load served as the dependent or criterion variable in this study, with the final model explaining 34.1% of its variance. Although 34.1% *R*^2^ is acceptable, it indicates that additional antecedents of cognitive load were not captured in the present model. Cognitive load is essential for interpreting, integrating, and deriving meaning from patient information during clinical decision-making^42,43^. Yet it must be carefully balanced—both insufficient and excessive cognitive load can impair decision-making performance^44–46^. Cognitive load also varies from patient to patient, influenced by case complexity, data density, and situational factors unique to each encounter. The present study could not account for these dynamic variations, nor could it examine how fluctuations in cognitive load affect clinical performance and how EHR design might adapt to these shifting cognitive demands.

Our findings suggest that improvements in both data usability and system usability are essential for achieving a balanced cognitive load, where unnecessary cognitive effort is minimized, and meaningful engagement with clinical information is supported. High data usability can encourage physicians to engage more deeply with patient information, yet poor system usability may redirect their attention toward navigating the interface, thereby increasing cognitive burden. These results underscore that cognitive load should be balanced rather than eliminated. High-quality, context-relevant data increase germane cognitive engagement, while well-designed system interfaces reduce extraneous cognitive effort. Together, these complementary improvements foster cognitively sustainable and error-resilient environments that enhance decision quality and support physician well-being. Our theoretical and empirical analysis further highlights that improvements in data usability can increase cognitive load. As data become more usable and therefore more diagnostically valuable, physicians naturally devote greater cognitive resources to interpreting them. Importantly, information overload decreases when physicians encounter highly usable patient data, as they are better able to focus more on task-relevant cues and filter out noise^47^. Conversely, improving EHR system usability reduces cognitive load by decreasing the mental effort required to operate the system. Currently, many EHR platforms remain difficult to use, forcing physicians to expend excessive cognitive effort on system interaction rather than clinical reasoning. In principle, data may be usable, and physicians may wish to focus on the information itself, but poor system usability diverts cognitive effort toward navigation and documentation, inflating cognitive load. Physicians should recognize that EHR systems inherently elevate cognitive load, often subtly, and these increases may influence clinical decisions. Future work will examine how fluctuations in cognitive load affect clinical decision-making performance and identify design strategies to mitigate these effects.

## Methods

**Fig. 3:**
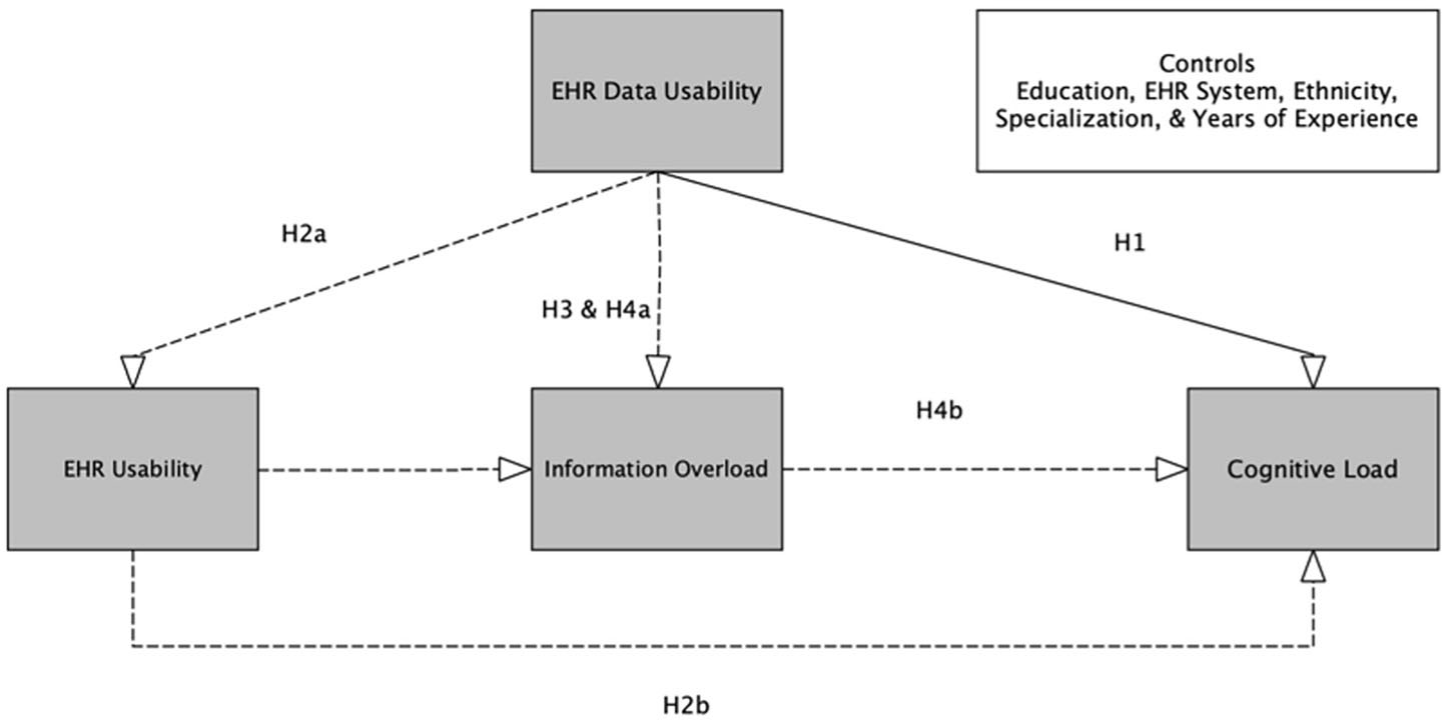
Hypothesized model of EHR data usability, system usability, information overload, and cognitive load. This figure depicts the hypothesized relationships among EHR data usability, EHR system usability, information overload, and physicians’ cognitive load. Solid arrows represent direct effects and dashed arrows represent mediated pathways. Control variables (education, EHR system, ethnicity, specialization, and years of experience) are included, but not the primary focus of the model.

We propose a research model outlining hypothesized relationships that explain the cognitive load physicians experience during EHR use, reference Fig. 3. The model addresses a key question: what explains a physician’s cognitive load during EHR use in a clinical setting^48^? Fig. 1 defines each construct, the expected direction of its associations, and the hypotheses to be empirically evaluated. The dependent variable is the physician’s cognitive load^7^. The independent variables reflect dimensions of EHR data usability: data completeness, data-driven use, and data quality. The mediating constructs are EHR system usability and physicians’ information overload. We tested the research model and its hypotheses using structural equation modeling (SEM).

*Cognitive load* occurs when physicians provide clinical care and make decisions. Cognitive load must be managed for effective problem-solving and clinical decision-making (correct decisions/reduction of errors), as levels of cognitive load influence decisions and outcomes^38,49–53^. In the context of EHRs and their effects, understanding how EHR data usability impacts physicians’ cognitive load is essential to understanding how EHR use affects clinical decision-making. With increased data use, higher-quality, evidence-based decisions, and greater access to EHR patient data, physicians are expected to analyze, diagnose, and treat problems more quickly. Generally, because EHRs have higher data usability prompts, they encourage physicians to engage more deeply with patient information^3,4,44,49,54^. This may increase cognitive load through germane (reasoning-related) effort. However, this effect should be distinguished from overload driven by poor system usability or workflow misalignment, which contribute to cognitive load^55^.

We posit:

H1: *EHR Data Usability has a direct and positive effect on Cognitive Load.*

Higher EHR data usability increases physicians’ cognitive load. When the cognitive load becomes too high, the quality of clinical decision-making declines. Cognitive load is reduced when EHR system workflows align with patient care routines and EHR systems are configured to minimize unnecessary distractions. Unnecessary factors (such as distractions) complicate use situations and raise cognitive load^38^. If the usability of the EHR system improves, it will reduce cognitive load. Therefore, when system usability better aligns with clinical workflow, physicians can focus more on medical tasks than on system usability, which decreases cognitive load. Therefore, improved EHR system usability will negatively mediate the positive effect of EHR system usability on cognitive load. Therefore, we posit:

H2: *EHR System Usability partially and negatively mediates the positive relationship between EHR Data Usability and Cognitive Load*.

As noted, in a clinical setting, information overload refers to excess environmental data, including all patient and situational data and their cues. Information overload increases (extraneous) cognitive load, impairing the physician’s ability to diagnose and make decisions. Continuous information overload leads to information fatigue, which in turn increases cognitive load. We posit that higher EHR data usability improves the accessibility and interpretability of clinical information, thereby reducing information overload by enabling physicians to focus on diagnostically relevant data rather than wasting effort sifting through irrelevant content. The use of the EHR system will direct physicians’ attention to system data and related aspects and decrease their attention to other information cues in the environment. Therefore, when EHR system usability improves, its effect can paradoxically reduce physicians’ cognitive load, as their attention becomes more focused on data examination and less on other cues in the environment. In this view, enhanced data usability mitigates overload not by limiting information volume, but by structuring and contextualizing data to reduce extraneous cognitive effort^47^. We posit:

H3: *EHR Data Usability directly and negatively influences information overload*.

When EHR data usability improves, physician cognitive load increases. Another way to reduce cognitive load is to minimize the physician’s attention to extraneous information in the environment and decrease information overload^56^. As EHR data properties improve, physicians can better filter out unnecessary data and information in their environment. Consequently, physicians become more focused on crucial aspects and use data to make evidence-based decisions. Environmental “noise” or unnecessary information is ignored. Therefore, physician’s focus increases because system focus is required. Increased focus lessens the perception of information overload, promotes attention economics, and helps physicians concentrate on specific environmental cues^38^. We posit that EHR data usability’s positive effect on cognitive load is also negatively mediated by information overload. EHR data usability decreases information overload and increases physician focus. We posit:

H4: *The positive effect of EHR data usability on cognitive load is negatively partially mediated by information overload*.

### Ethics

The study protocol was reviewed and approved by the Case Western Reserve University Institutional Review Board (IRB) as an Exempt Human Subjects Research protocol under federal category 45 CFR 46.104(*d*)(2), which covers survey-based research that does not collect identifiable private information and in which disclosure would not place participants at risk. The protocol was approved under reference Merriweather_HRP-503EXEMPTC_11-5-2021, with an approval letter issued on November 11, 2021, at 5:12 PM. As this was a single-site protocol, no additional local ethics committees or IRBs were involved. All participants provided informed consent via an electronic information sheet prior to beginning the survey, and all study procedures adhered to the principles of the Declaration of Helsinki.

### Study design

This study used a cross-sectional survey design to assess how EHR system usability and data usability contribute to physicians’ cognitive load during clinical decision-making. Participants received an email invitation that included the study description and consent information; those who agreed completed an online questionnaire that assessed EHR data usability, EHR system usability, information overload, and cognitive load. Item order was randomized to minimize common method bias. Participation was voluntary, and no financial incentives were offered.

### Participants and recruitment

Data were collected via an online Qualtrics survey administered between February and May 2022. Participants were recruited through SBH Global, and eligibility was confirmed using screening questions. Eligible participants were practicing physicians with a minimum of 4.84 years of EHR experience. Medical trainees and physicians without direct patient care responsibilities were excluded. Respondents had to actively use an EHR and be employed as a physician for a healthcare organization within the United States.

Since the goal was to examine the cognitive load antecedents of physicians using EHR systems, we excluded billing administrators, healthcare executives, informatics, and nurses from the sample. Participants were licensed physicians practicing in the United States across multiple specialties and healthcare systems.

### Measures and variables

We treated all first-order constructs as reflective, latent constructs and used a five-point Likert scale for all first-order items. All scales were adopted from published validated measures and adapted to reflect the physician’s setting and perspective.

#### Cognitive load

Cognitive load refers to the amount of working memory resources available and how much they are used^17^. We used Lu et al.’s Physician Mental Workload Scale^57^. The scale captures dimensions of cognitive activities, multiple tasks, and overcoming cognitive difficulties as part of cognitive load. The scale consists of three items reflecting aspects such as: “cognitive load increases when performing multiple tasks,” “frequent clinical alerts increase cognitive load,” and “administrative load increases cognitive load.” Two items were deleted for low loading (<0.40) and cross-loading. We used three remaining items with good reliability (*α* = 0.79).

#### EHR data usability

EHR data usability refers to the extent to which data within the EHR are of sufficient quality, completeness, and contextual relevance to support accurate and efficient clinical decision-making. This construct reflects physicians’ perceptions of whether the information presented is reliable, current, and appropriate for the clinical task at hand. In this study, data quality, data completeness, and data-driven use were modeled as first-order indicators that contribute to the second-order latent factor of EHR data usability. These dimensions were not analyzed separately in the final structural model; instead, they were combined to form a higher-order construct. To operationalize this construct, we modeled a second-order latent factor comprising three first-order dimensions: data completeness, data-driven use, and data quality. The final scale included ten items, which loaded onto a single factor with acceptable internal reliability (*α* = 0.88).

#### Data completeness

Data completeness refers to the extent to which needed data is available for analysis or other tasks^58^. We used the scale by Liu et al.^58^, which measures data completeness within EHR records. The scale includes three items, such as “electronic health record (EHR) patient data is complete” and “patient care is better with the electronic health record (EHR).” Two items were deleted due to low loading (<0.40) and cross-loading. We used the remaining three items with good reliability (*α* = 0.86).

#### Data-driven use

Data-driven use refers to a process or activity motivated by data rather than mere intuition or personal experience. Decisions are based on empirical evidence and not speculation or gut feeling^59^. We drew from the scale by Yu et al.^59^ which captures the data-driven use of systems in hospital operations. The scale consists of two items: “data is more important than personal instinct” and “evidence-based physicians transform data into information.” Three items were deleted for low loading (<0.40) and cross-loading. We used the two remaining items with good reliability (*α* = 0.70).

#### Data quality

Data quality refers to the accuracy of information retrieved from EHR systems. High-quality data means data that is defect-free^60^. We used the scale developed by Lev^12,60^, who measured information quality in EHR systems. The scale consists of five items, such as “EHR patient data has high quality,” “physicians accurately record patient data,” and “I assume errors may exist in recorded clinical data.” We used all five items with good reliability (*α* = 0.87).

#### EHR usability

EHR usability is defined as how user-friendly, useful, and likable a system is for its clinical users when performing medical tasks^61^. It also indicates how well the EHR aligns with patient care processes. The system usability measure reflects physicians’ perceptions of the EHR’s UI, its navigation, responsiveness, and alignment with clinical workflow, rather than technical architecture. The data usability construct captures the perceived completeness, accuracy, and contextual relevance of patient data within the EHR. We adapted the scale by Shultz and Hand^62^, which measures EHR usability within clinical workflow. The scale consists of three items: “EHR workflow aligns with routine care processes,” “EHR tasks are well aligned with current job role,” and “EHR functionality is well integrated with clinical tasks.” Two items were deleted for low loading (<0.40) and cross-loading. We used three remaining items with good reliability (*α* = 0.89).

#### Information overload

Information overload refers to an individual’s inability to manage excessive incoming information over time, which may lead to increased stress or even burnout. We adapted the scale by Weber et al.^63^, which captures information overload. The scale includes five items, such as: “I have a difficult time sifting through the overabundance of patient information within EHR” and “due to the volume of information in EHR, I have difficulty finding the patient information needed”. Three items were removed due to low loading (<0.40) and cross-loading. We used the two remaining items, which demonstrated good reliability (*α* = 0.82).

#### Physician characteristics (controls)

Physician characteristics, including education, ethnicity, gender, medical specialization, and years of experience, were collected as control variables to account for potential demographic and experiential influences on information overload and cognitive load. Preliminary analysis indicated that none of these variables had significant direct effects on the dependent constructs; therefore, they were excluded from the final structural model to maintain parsimony.

#### Social desirability (quality check)

Social desirability refers to a participant’s tendency to give “desirable” answers to create a more socially acceptable self-image^64^. To address this potential bias, we included a validated five-item social desirability scale^64^. Two items were removed due to cross-loading and weak factor loadings (< 0.40), leaving a reliable three-item scale (*α* = 0.80). This construct was used solely as a measurement quality check to confirm response integrity and assess potential bias. As no substantial variance inflation or patterned response bias was observed, the variable was not retained in the final structural model.

### Instrument development

A 19-item Qualtrics survey instrument was developed for this study. The survey employed validated scales adapted from prior research in health informatics and cognitive psychology. We validated items for conceptual clarity and meaning through 9 rounds of Q-sorts with 12 practitioners. Q-sorts were conducted until a hit rate of 75% or higher was achieved to establish each item’s meaning and consistency (Thomas et al.^65^). Each item that failed to reach a hit rate of 70% or higher was re-examined, condensed for clarity, and reworded to reduce cognitive processing threats. A total of 146 item changes were made to sentence structure to achieve a hit rate of 75% or higher for each Q-sorted item. Attention was given to respondents’ understanding of the distinction between decision-making, EHR data usefulness properties, and joint sensemaking constructs. Once Q-sorting was finalized, we conducted pilot testing. The average response time was 12:53 min, with an outlier of 20 min. The outlier skewed the sample and was removed, resulting in an acceptable average survey completion time (<8 min).

### Data collection procedures

Conceptual definitions of all constructs are described in the Model Development section. Each construct was operationalized using validated scales adapted from prior research, with items rated on a 5-point Likert scale (1 = strongly disagree to 5 = strongly agree). Internal reliability and factor loadings met established thresholds.

### Data screening and preparation

#### Measurement model

Twenty-six records were removed due to missing data, unengaged responses, or outliers on continuous variables. Cases with more than 5% missing data were excluded. An additional 23 records were removed because respondents did not report active EHR system use. For the remaining dataset, 20 missing values were imputed using the median value of the corresponding scale^31,66^. Homogeneity of variance was confirmed across all constructs and items. Six items demonstrated acceptable skewness (<|1.1|), and 10 items demonstrated acceptable kurtosis (<|2.2|), consistent with recommended thresholds (Hair et al.^31^).

### Data analysis

Data were analyzed using covariance-based SEM in Mplus (version 1.8.7) with maximum likelihood estimation. Measurement model evaluation included assessment of factor loadings, Cronbach’s alpha, CR, and AVE. We conducted an exploratory factor analysis (Maximum-Likelihood extraction with Direct Oblimin rotation) to identify the factor structure and remove items with low loadings or problematic cross-loadings. We then performed confirmatory factor analysis to compare first-order and second-order specifications and to verify convergent and discriminant validity.

Structural model evaluation included examination of path coefficients, p-values, and *R*^2^ values for endogenous constructs. Overall model fit was assessed using χ²/df, CFI, TLI, RMSEA, PCLOSE, and SRMR. Indirect effects for mediation hypotheses were tested using bootstrapping with 5,000 resamples and 95% confidence intervals. To assess common method bias, we incorporated a CLF following Podsakoff et al. and inspected changes in fit. Multicollinearity was examined using VIFs. Control variables (age, gender, specialty, years of experience, etc.) were initially included as covariates, but because none showed significant effects, they were removed from the final model for parsimony. The study was reported in accordance with STROBE guidelines for cross-sectional research.

## Data Availability

The de-identified dataset generated and analyzed during the current study is available from the corresponding author upon reasonable request. Data are shared for academic research use only and in accordance with institutional ethical approval.

## Code availability

All structural equation modeling (SEM) syntax used in the analysis (Mplus v1.8.7) is available from the corresponding author upon reasonable request.
